# Antibiotic prescribing behavior among physicians: ethical challenges in resource-poor settings

**Published:** 2018-05-12

**Authors:** Saurav Basu, Suneela Garg

**Affiliations:** 1 *Junior Resident, Department of Community Medicine, Maulana Azad Medical College, New Delhi, India. *; 2 *Director Professor, Department of Community Medicine, Maulana Azad Medical College, New Delhi, India.*

**Keywords:** *Ethics*, *Antibiotics*, *Antimicrobial resistance*

## Abstract

Prescribing antibiotics to patients represents an ethical dilemma for physicians since the current health needs of the patients have to be balanced with concerns for long term containment of antimicrobial resistance in the community. Overuse of antibiotics is a major pathway for development of antimicrobial resistance. In resource-poor settings a complex social reality can influence antibiotic prescribing behavior among physicians which apparently violates the conventional biomedical ethics principles especially beneficence and justice. These social factors include patient socioeconomic class, patient demand for antibiotics, competition among practitioners and conflict of interest arising from the physician’s social relationship with his/her patient. Current approaches for combating antimicrobial resistance in the developing countries are inadequate in factoring and dealing with those irrational prescription practices which are driven predominantly by subtle violation of medical ethics as opposed to blatant economic and professional profiteering.

## Introduction

Prescription of antibiotics presents an ethical dilemma for physicians since the current health needs of patients have to be balanced with concerns for their safety due to adverse effects and restricting antimicrobial resistance (AMR) throughout the community (1 - 2). The alarming increase in AMR is a major public health challenge worldwide (3). Overuse of antibiotics is a major pathway for development of AMR, which adversely impacts population health by increasing mortality and disability rates due to infectious diseases, escalating treatment costs and reduction of economic productivity (4). As no new classes of antibiotics have been developed in the past 2 - 3 decades, antibiotics represent a scarce resource that should be used in a sustainable manner in order to protect the health needs of future generations (1, 5). Therefore, promoting judicious use and avoiding irrational prescription of antibiotics is considered a key strategy for containing AMR in communities. Factors like financial incentives from pharmaceutical companies and lack of knowledge about antibiotic prescription guidelines can lead to erroneous antibiotic prescribing practices. However, it could be argued that there exists a complex social reality underlying the antibiotic prescribing behavior that apparently violates the conventional biomedical ethics principles of patient autonomy, beneficence, non-maleficence and justice. Some of these situations in mostly resource-poor outpatient settings are discussed below:

*Clinical judgment influenced by patients’ socioeconomic status*

While prescribing antibiotics, especially in resource-poor settings, physicians often have to rely upon clinical evaluation alone in the absence of adequate diagnostic support. Empirical therapy is not always unwarranted; however, the high cost of investigations like blood/urine culture and sensitivity testing, which are not affordable or easily accessible for poor patients, can influence the physician’s behavior (6). Similarly, even in developed countries, physicians are more likely to prescribe antibiotics to socioeconomically deprived patients due to concerns about complications (7), which may actually arise from poor hygiene, sanitation and nutrition (6). Nevertheless, this mode of antibiotic prescription may be an example of what is socially just but ethically unacceptable since the health of future generations can be compromised through potentially increased antibiotic resistance resulting from empirical therapy. The physician here decides that the benefit incurred to the patient through empirical therapy outweighs the consequences of potential increased antibiotic resistance or environmental degradation in the future (8), although this decision is not evidence based.

*Patient demand for antibiotics*

There is global evidence that physicians can be pressured into prescribing antibiotics even against their clinical judgment (7). Patients can employ a variety of both direct and indirect measures in this process. Direct demands for antibiotics may arise from patients’ expectation of medicine during their very first visit even in the absence of investigation. In most developing countries like India, it has been observed that intense competition between private practitioners can distort physicians’ prescribing practices. The reason is the concern that the patient may not turn up again (9) and instead get antibiotic medications anyway from another practitioner or even directly from a pharmacist. Indirect demands for antibiotics have been reported worldwide and include patients enquiring and citing past instances of medical improvement after taking antibiotics (7). Consequently, such actions can result in prescription of antibiotics when they are not required at all, as in the case of viral illnesses. The result will be a compromise of beneficence for the patient and long-term promotion of antibiotic resistance in the environment and community. 

*Conflict of interests and bias*

Even during unavoidable empirical therapy, narrow-spectrum antibiotics should be prescribed as opposed to broad-spectrum antibiotics, even though nonclinical and social factors can influence the type of antibiotic prescribed. It is ethically appropriate for physicians to write prescriptions for their family members to cure short-term minor problems, that is, if they possess the necessary expertise to treat the condition (10). Nevertheless, such assumptions do not cater to the possibility of ethical conflicts arising from flawed antibiotic prescription. While prescribing antibiotics to their family, friends or influential patients, physicians may tend to prescribe the “better” antibiotic, which is likely to be broad-spectrum or one against which little resistance has been reported. This is because prescription of a narrow-spectrum antibiotic for empirical therapy inevitably involves the risk of lack of benefit, especially in communities where prevailing AMR is high. Consequently, a physician anticipating potential failure of narrow-spectrum antibiotic therapy and concerned with the risk of prolonged sickness in those with whom he or she is emotionally attached may prescribe broad-spectrum antibiotics. A physician who adheres to a rational prescription of antibiotics to patients who are strangers may therefore fail to do so with patients with whom he or she shares an intimate relationship. This kind of differential treatment is unethical since it often follows a distinct class bias as affluent patients are more likely to exert bargaining power upon a physician compared to those who are economically weak. Moreover, such practice violates the ethical principle of justice, which requires fair distribution of scarce resources. 

In developing countries, current approaches to containing AMR focusing on prescription audits, sensitization and training of physicians tend to rely upon clinical worldviews in which pharmaceutical incentives and the urge for gratifying patient expectations preclude rational antibiotic prescribing (11 - 12). Nevertheless, there is a need to distinguish between the following antibiotic prescription practices: 1) an irrational approach guided by blatant economic and professional profiteering, and 2) one involving a subtle ethical violation with the intention to overcome existing clinico-social challenges. Public policies for containing AMR in developing countries focusing upon promotion of rational drug prescription need to envisage best practices for physicians in outpatient settings when confronted with such ethical dilemmas and challenges in treating their patients.
